# Illegal intra-corporeal packets: can dual energy CT be used for the evaluation of cocaine concentration? A cross sectional study

**DOI:** 10.1186/s12880-016-0106-3

**Published:** 2016-01-13

**Authors:** Alexandra Platon, Minerva Becker, Christoph D. Becker, Eric Lock, Hans Wolff, Thomas Perneger, Pierre-Alexandre Poletti

**Affiliations:** Department of Radiology, University Hospital of Geneva, 4, rue Gabrielle-Perret-Gentil, 1211, Geneva, Switzerland; Scientific Police, Geneva State Police, Geneva, Switzerland; Division of Correctional Medicine and Psychiatry, University Hospital of Geneva, ch. du Petit-Bel-Air 2, 1225, Chêne-Bourg, Switzerland; Division of Clinical Epidemiology, University Hospital of Geneva, 4, rue Gabrielle-Perret-Gentil, Geneva, Switzerland

**Keywords:** Computed tomography, Dual energy body packing, Cocaine

## Abstract

**Background:**

The recent implementation of the dual energy technology on CT-scanners has opened new perspectives in tissue and material characterization. This study aims to evaluate whether dual energy CT can be used to assess the concentration of cocaine of intra-intestinal illegal packets.

**Methods:**

The study was approved by the institutional review board of our institution (CER 13_027_R). From November 2010 to May 2013, all consecutive conveyors in whom a low-dose abdominal CT (LDCT) revealed the presence of illegal intra-corporeal drug packets underwent a dual energy CT series (gemstone spectral imaging) targeted on one container. The mean radiological density (HU) of these packets was measured on the LDCT series, and on the monochromatic dual energy series, at 40 and 140 keV. The difference between the HU at 40 and 140 keV was reported as ∆HU. The effective atomic number Z(eff) was also measured on the monochromatic series. A chemical analysis was performed after expulsion to select cocaine containing packets, and to determine their cocaine concentrations. A correlation analysis was performed between HU, ∆HU and Z(eff), with regard to the percentage of cocaine.

**Results:**

Fifty-four cocaine conveyors were included. The mean cocaine content of the packets was 36.8 % (range 11.2–80, SD 15.4), the mean radiologic density 105 HU, the mean Z(eff) 8.7 and the mean ∆HU 163. The cocaine content was correlated with the ∆HU (0.57, *p* < 0.001), with the Z(eff) (r = 0.56, *p* < 0.001) but not with radiologic density (r = 0.25, *p* = 0.064). ∆HU >200 was 0.9 (9 of 10) sensitive and 0.82 (36 of 44) specific to predict a cocaine concentration higher than 50 %.

**Conclusion:**

Measuring ∆HU or Z(eff) on dual energy monochromatic CT series can be used to detect ingested packets with cocaine concentration >50 %.

## Background

Body packing refers to the act of concealing large drug containers (usually >2 cm) within the gastro-intestinal tract. Cocaine and heroin are the most frequent illicit substances smuggled through customs in this way. Standard radiography is often used as a screening test for the detection of intracorporeal body packets, but it is limited by a relatively high rate of false negative interpretations when compared to low-dose CT (LDCT) imaging [1–5]. Therefore, LDCT is now progressively replacing standard radiography in the screening of the persons suspected of conveying body packets. Furthermore, once body packets have been identified, a follow-up LDCT gives also useful information for the detection of potential complications up to the complete evacuation of the packets, such as the absence of progression of a packet through the gastro-intestinal tract, thus increasing the risk of packet rupture [6]. However, it has never been established whether LDCT might bring information about the packets content, which could be clinically relevant in case of impending rupture. The recent implementation of the dual energy technology on CT-scanners has opened new perspectives in tissue and material characterization [7]. Recent in vitro series have suggested that dual energy CT technology (DECT) may be used to differentiate heroin from cocaine in illegal intra-corporeal packets, swallowed by drug smugglers [8, 9], but no studies were performed while packets were still in the gastro-intestinal tract. In our institution, almost all illegal packets contain cocaine, which is due to the specificities of the international drug trafficking pathways. In this setting, knowing the concentration of cocaine contained in the packets may be of great interest for the clinician in charge of the drug conveyer to adapt the therapeutic management in case of impeding rupture.

The aim of the current study is to evaluate whether dual energy CT can be used to assess the concentration of cocaine of intra-intestinal illegal packets.

## Methods

The research project was approved by the Institutional Review Board of our institution (Ethics Committee on research involving humans of the University Hospital of Geneva, CER 13_027_R), in compliance with the Helsinki Declaration. Since a better characterization of the packets by a targeted DECT series was considered useful to improve the clinical management of the conveyors, and therefore their safety, the Ethics committee waived the need for consent.

All consecutive adult persons suspected of having ingested drug packets within the Geneva State territory (*n* = 720) during a 30 months period of time (November 2010 to May 2013) were brought to our emergency department for a LDCT examination. LDCT images were immediately interpreted for the presence of drug containers by the radiology resident and the attending physician on call, while the suspect was still on the CT table.

Whenever LDCT was reported positive for body-packets (*n* = 120, 16.5 %), a 3-cm thick CT series was performed, targeted on a drug container, using a dual-energy protocol (Gemstone imaging, GSI).

When the presence of body-packets was reported at LDCT, the conveyors were hospitalized until all packets were collected. For each patient, four of these packets were analyzed by a dedicated laboratory, which evaluated their size and weight, the percentage of cocaine and also performed a qualitative analysis of the cutting agents. These data were retained by the scientific police up to the end of the study.

### Exclusion criteria

Cases were excluded from the study when laboratory data could not be obtained or when dual energy series could not be performed. When the cocaine concentrations measured in various packets from the same conveyor were dissimilar (variation of more than 20 % between the highest and the lowest drug concentration), the case was also excluded.

### Reference standard

The mean percentage of cocaine obtained from the 4 measures from samples of homogenous drug packets was considered reference standard.

### Technical imaging parameters

LDCT were performed with a 64-rows GE 750 HD CT (Discovery 750 HD CT, GE Healthcare, Milwaukee, USA), from lung bases to pelvis, without intra-venous, oral or rectal contrast material, using the following parameters: 64 ×1.25 mm collimation, pitch 1.375, gantry rotation period 0.7 s, tube potential 120 kV, tube charge per gantry rotation 25.2 mAs, reconstruction slice thickness 2.5 mm, using 40 % ASIR (adaptive statistical iterative reconstruction).

GSI series were performed on the same CT scanner as LDCT, on a 3 cm thick area, using the following parameters: 64 ×1.25 mm, pitch 1.375, 140 kV, gantry rotation period 0.6 s, variable potential output of 80 kV and 140 kV, tube charge per gantry rotation 180 mAs.

### Effective dose of LDCT and of GSI series

The dose of radiation delivered by low-dose CT and the dual energy series were estimated from the mean normalized values of effective dose per dose-length product (DLP) for the abdomen, as described by Shrimpton et al. [10]. The effective dose for LDCT was 1.4 mSv (DLP = 94.15 [mGy.cm]) for men, and 1.2 mSv for women (DLP 83.54 [mGy.cm]). For the dual energy series, the effective doses were 1.27 mSv and 0.9 mSv for men and women respectively.

### CT images analysis

LDCT images and dual CT data were transferred and analyzed on a dedicated GE Advantage workstation (ADW, GE Healthcare, version 4.6), using a GSI viewer 2.0-2L.

**Fig. 1:**
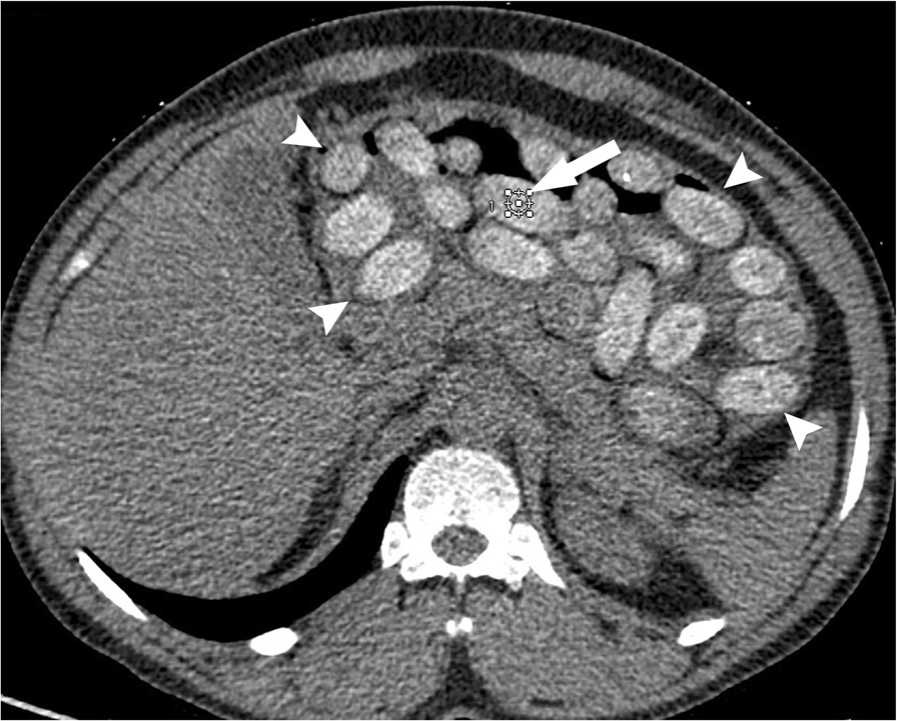
Thirty-years-old man, positive for conveying dug packets. Axial low-dose CT scan shows numerous drug containers inside the stomach (*arrowheads*); measurement of attenuation is performed within one drug container (*arrow*)

**Fig. 2:**
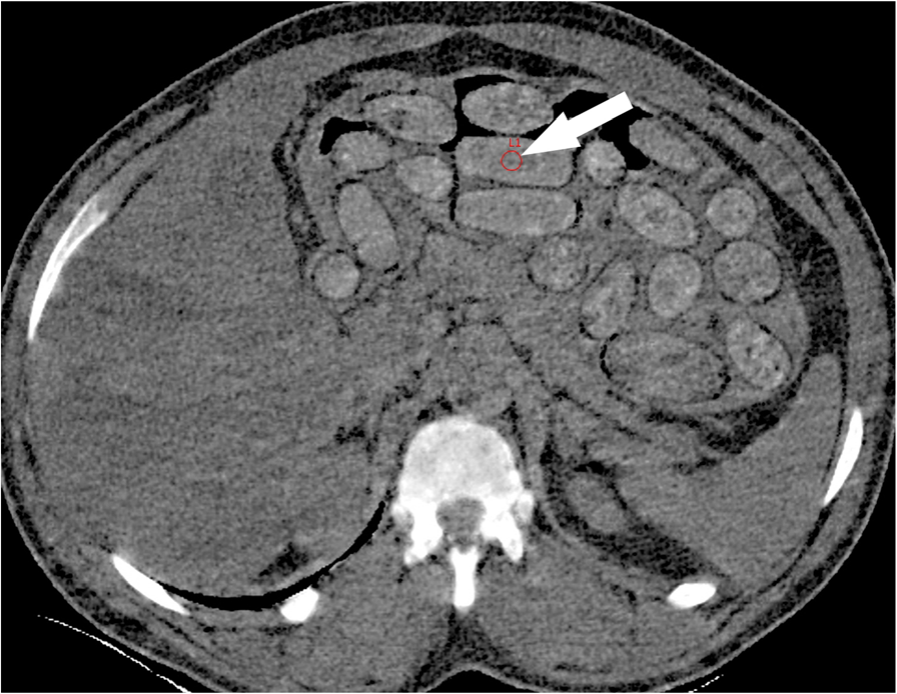
Thirty-years-old man, positive for conveying dug packets. Dual-energy CT scan shows measurement of the Z(eff) within the same drug container (*arrow*)

LDCT and dual-energy CT images were prospectively analyzed by one of the two board certified attending radiologist of the emergency radiology unit, with 15 and 17 years of experience respectively in reading abdominal CTs. Both radiologists were blinded to the results of the chemical analysis of the packets. The radiologist measured the mean density (HU) in a packet on the LDCT series, using a relatively small (60 mm^2^) circular region of interest (ROI), to reduce the risk of partial volume averaging (Fig. 1). The same packet was selected within the 3 cm thick GSI series; the mean effective atomic number Z(eff) (Fig. 2), as well as the mean density (HU) at 40 and 140 keV, were measured in a 60 mm^2^ circular ROI. The difference between the attenuations at 40 and 140 keV was calculated and referred to as ∆HU.

### Statistical analysis

We obtained mean values and standard deviations of all continuous variables. Then we examined associations between cocaine content (expressed in percent) and various measures of radiologic density using scatterplots and summarized them through Pearson correlation coefficients.

Since the median packet weight in our study population is 10 g, a cocaine content of 35 % or higher is close to the reported lethal threshold (about 3 to 4 g) in case of packet rupture. Therefore, we dichotomized cocaine content as below or above 35 %. We also dichotomized cocaine content as below or above 50 %, to focus on packets with major lethal risk in case of rupture. Then we examined associations between the cocaine concentration at these thresholds with measures of radiologic density by means of receiver operating characteristic curves (ROC) and corresponding areas under the curve (AUC). An AUC of 0.5 means no association while an AUC of 1 reflects perfect discrimination between low-content and high-content containers.

For the most promising predictor, we looked at cutoff values that achieved a good compromise of sensitivity and specificity, and reported statistics of test performance.

Analyses were performed using SPSS version 18 (PASW Statistics 18, SPSS Inc, Chicago, USA).

## Results

Dual energy CT-scans were not available for 22 of the 120 conveyors in whom drug packets were detected at LDCT; these cases were removed from the study. Forty-four of the 98 remaining cases had to be further excluded, because the laboratory analysis could not be obtained (*n* = 26) or because packets were dissimilar (*n* = 18).

Thus, our study population consisted in 54 conveyors (45 men, 9 women), with a mean age of 34.5 years (range 20–54). The mean amount of packets per conveyor was 34 (range 4–70). The cocaine content ranged from 11 to 80 %, with a mean of 36.8 % (Table 1). The cocaine content was ≤35 in 30 conveyors, >35 % in 24 conveyors, and >50 % in 10. The weight of the packets ranged from 9.6 to 11.8 g (mean 10.04 g).

**Table 1:** Radiological analyses of packets cocaine content. Means, standard deviations (SD), and correlations between cocaine content, measures of radiologic density (HU) and effective atomic number Z(eff), in 54 drug containers

Variable	Mean (standard deviation)	Pearson r	*P* value
Cocaine content (%)	36.8 (15.4)	–	–
HU low dose CT	105.4 (94.9)	0.25	0.064
Z(eff)	8.71 (0.82)	0.56	<0.001
HU 40 keV	236.7 (174.3)	0.42	0.001
HU 140 keV	73.3 (82.5)	−0.01	0.95
∆HU^a^	163.4 (130.8)	0.57	<0.001

^a^∆HU = difference between attenuation at 40 keV and the corresponding 140 keV value

The most frequent cutting substances detected included phenacetin (49 containers, 90.7 %), levamisole (47, 87.0 %), sugars (38, 70.4 %), lidocaine (34, 63.0 %), caffeine (34, 63.0 %), and mannitol (33, 61.1 %).

### Associations with radiologic density measures

Cocaine content was moderately associated with Hounsfield units (HU) at 40 keV, but not with HU at 140 keV. However, the difference between these values (∆HU) showed a very strong correlation with the cocaine content. The results were similar for the Z(eff). Measures of the densities based on the low dose CT scan were only weakly associated with cocaine content (Table 1).

**Fig. 3:**
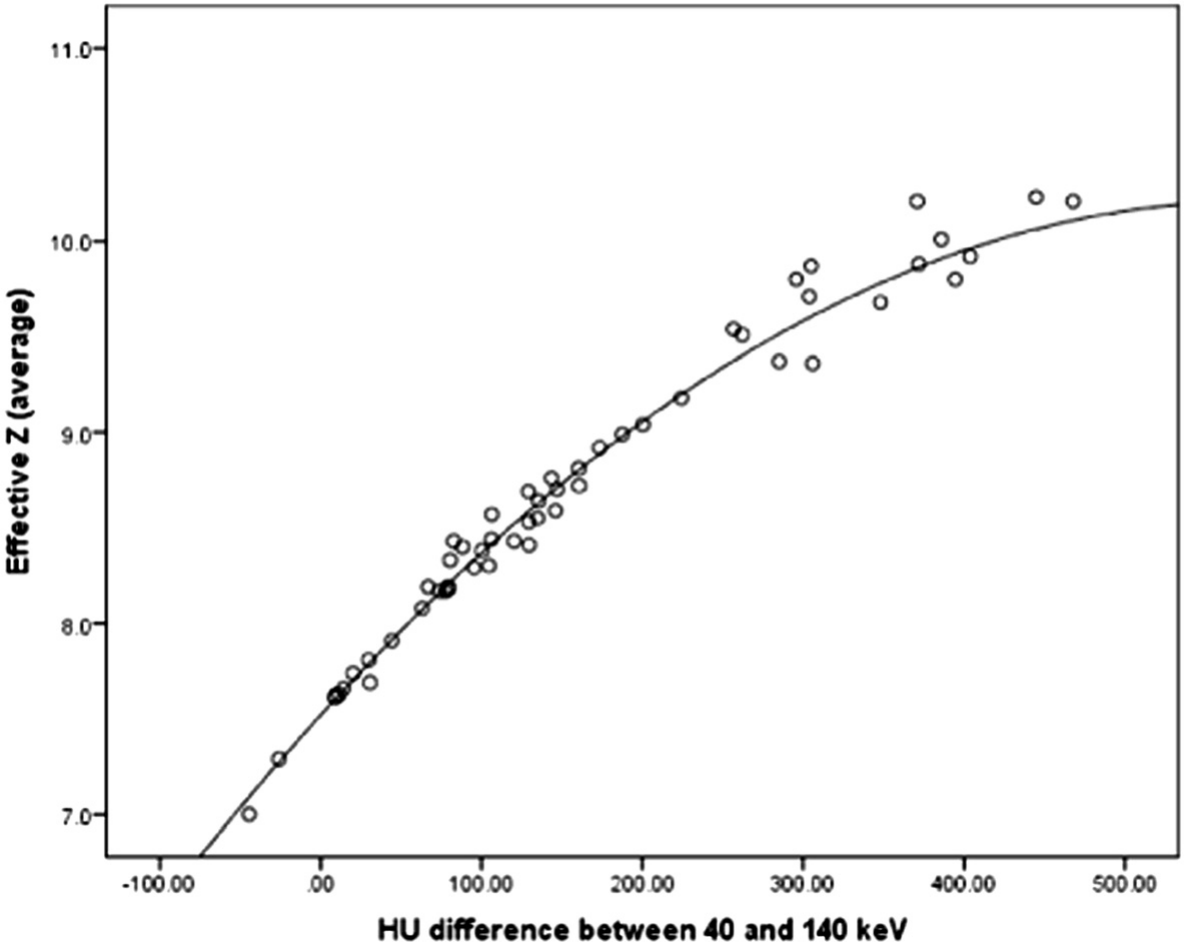
Scatter-plot of the effective atomic number Z(eff) versus ΔHU (r = 0.98). Z(eff) can be inferred from ∆HU using the equation of the curve: Z(eff) = 7.521 + ∆HU × 0.009275 − 0.000008 × ∆HU^2^

We also confirmed that the ∆HU and the Z(eff) contained practically identical information; the Pearson correlation between these 2 variables was 0.98. Because the method for computing the Z(eff) is proprietary, and therefore only available on a GE system, we displayed our results in ∆HU values only. To obtain the corresponding Z(eff) value from ∆HU, we established a correspondence curve (Fig. 3). The equation of this curve is: Z(eff) = 7.521 + ∆HU × 0.009275 − 0.000008 × ∆HU^2^.

**Fig. 4:**
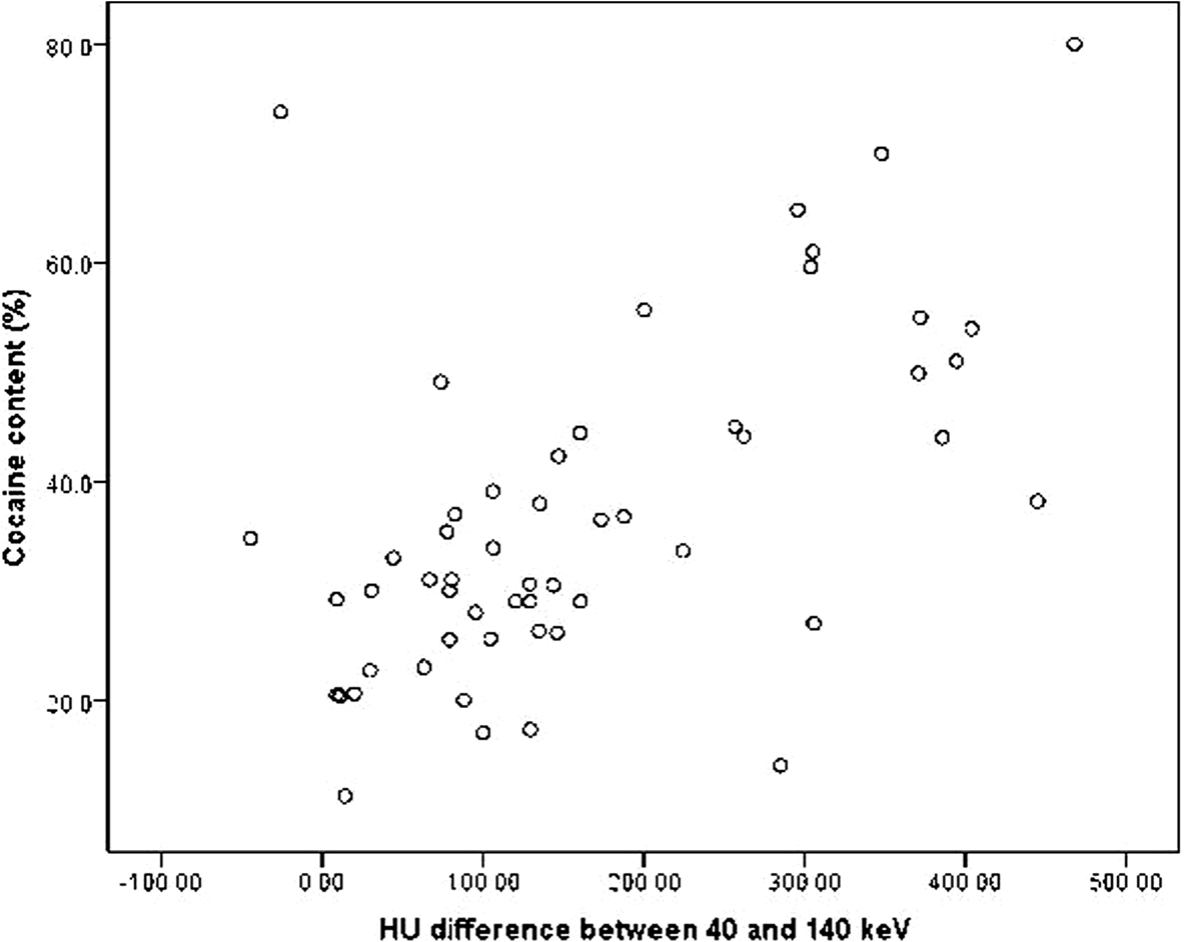
Scatter-plot of cocaine content versus ΔHU

**Fig. 5:**
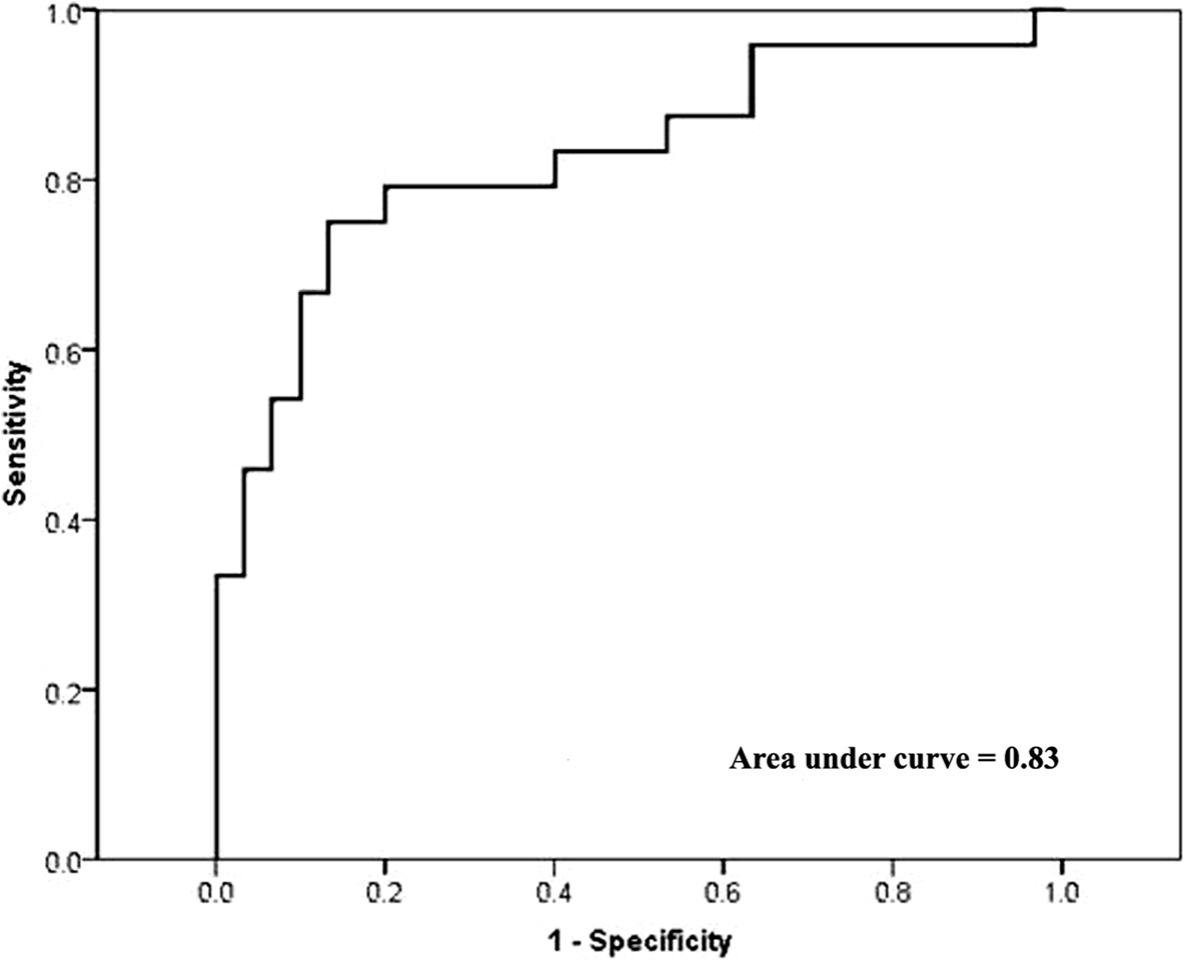
Receiving operating characteristic curves for ΔHU versus cocaine content >35 %

**Fig. 6:**
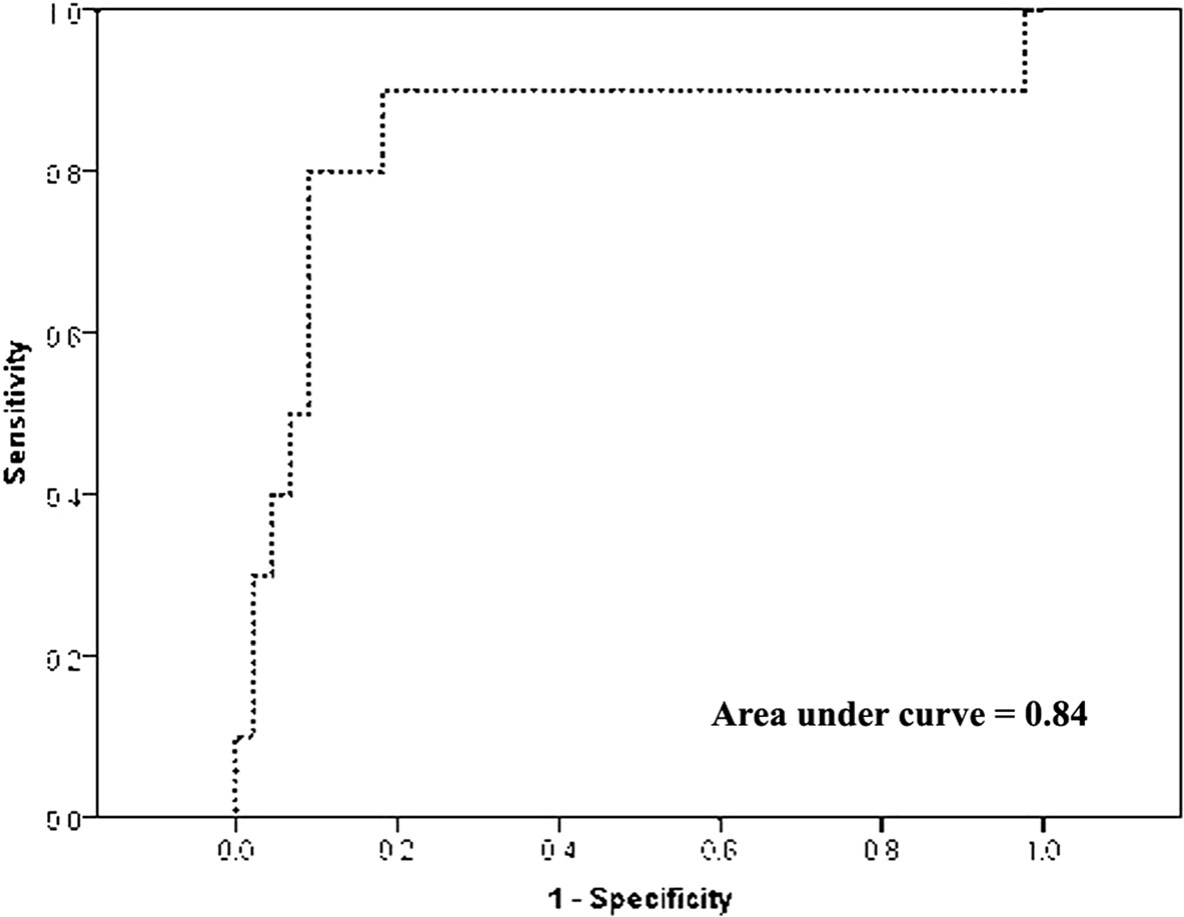
Receiving operating characteristic curves for ΔHU versus cocaine content >50 %

The association between ∆HU and cocaine content was continuous, with no evidence of a threshold (Fig. 4). The same pattern of associations was seen when cocaine content was dichotomized at 35 and 50 %. The areas under the ROC curves were 0.83 (95 % CI 0.71 to 0.94) for cocaine content dichotomized at 35 % (Fig. 5), and 0.84 (95 % CI 0.62 to 1.00) for cocaine content dichotomized at 50 % (Fig. 6). ∆HU achieved better sensitivities and specificities for detecting packets with very high cocaine content (>50 %) than for detecting packets with high cocaine content (>35 %), (Table 2).

**Table 2:** Diagnostic performance of ∆HU for 2 levels of cocaine content

Variable	Sensitivity	Specificity	Positive predictive value	Negative predictive value	Odds ratio (95 % confidence interval)
Cocaine content >35 %					
∆HU >130	0.79 (19/24)	0.77 (23/30)	0.73 (19/26)	0.82 (23/28)	12.5 (3.4–45.7)
∆HU >140	0.75 (18/24)	0.80 (24/30)	0.75 (18/24)	0.80 (24/30)	12.0 (3.3–43.4)
∆HU >150	0.71 (17/24)	0.87 (26/30)	0.81 (17/21)	0.79 (26/33)	15.8 (4.0–62.3)
Cocaine content >50 %					
∆ HU >200	0.90 (9/10)	0.82 (36/44)	0.53 (9/17)	0.97 (36/37)	40.5 (4.5–366.8)
∆ HU >250	0.80 (8/10)	0.84 (37/44)	0.53 (8/15)	0.95 (37/39)	21.1 (3.7–121.4)
∆HU >300	0.70 (7/10)	0.91 (40/44)	0.64 (7/11)	0.93 (40/43)	23.3 (4.3–127.6)

∆HU: difference between attenuation at 40 keV and the corresponding 140 keV value

## Discussion

The data from the current studies suggest that DECT can be used to estimate the concentration of cocaine contained in an intracorporeal packet, before its expulsion. DECT achieved a good sensitivity (79 %) and specificity (77 %) for detecting packets with >35 % cocaine concentration, and an even better sensitivity (90 %) and specificity (82 %) to identify packets with very high cocaine content (>50 %). This improvement in sensitivity could be explained by the fact that the packet content is more homogenous when the percentage of cocaine is higher and, thus, the percentage of cutting agents smaller. Indeed, laboratory analyses of the current study revealed that cocaine was almost always mixed with multiple cutting agents, varying in quality and quantity from one conveyor to the other. Besides, the number of these cutting agents has probably been underestimated, since it is impossible for a laboratory to perform an exhaustive evaluation of all potential substances that may be contained in a packet. Therefore, the influence of the cutting agents on DECT imaging is unpredictable, especially when the percentage of cocaine is low, which certainly constitutes a limitation of the methodology.

Another relevant observation is the linear relationship between ∆HU and Z(eff), and the fact that ∆HU is equivalent to the Z(eff) measures to predict the packet’s cocaine concentration. Unlike ∆HU, the algorithm used to measure Z(eff) is proprietary and, therefore, not communicated by the manufacturer. ∆HU, however, can be easily obtained on any dual-energy CT equipment. We therefore recommend using this parameter when reporting results in the scientific literature.

The third observation of the current study is the fact that the cocaine concentration of a packet cannot be assessed by measuring the HU on the standard LDCT images. This can be explained by the fact that the density of the packets varies with the degree of drug compression, as suggested by prior series [1, 8], while the difference in attenuation by DECT, however, would remain unaffected by the compression [8, 9, 11].

Two prior in-vitro series suggested that DECT may be useful to differentiate heroin from cocaine [8, 9]. Since our conveyors population mainly consisted in cocaine smugglers, it was not possible to compare these prior in-vitro qualitative reports with our in-vivo quantitative observations. In a population of cocaine smugglers, the assessment of the percentage of the drug in the packets before expulsion could certainly play a role in the choice of the most appropriate treatment, in case of rupture or impending rupture.

## Conclusion

In conclusion, our study showed that DECT may be used for assessing the concentration of cocaine in intracorporeal containers, while the packets are still in the gastrointestinal tract of drug conveyors. Further in vitro series are mandated to increase the precision of the measures by a better understanding of the influence of the cutting agents on DECT results.

## Abbreviations

LDCT: Low-dose CT
DECT: Dual energy CT
GSI: Gemstone imaging
Z(eff): Mean effective atomic number
HU: Hounsfield Unit
∆HU: Difference between the attenuations at 40 keV and 140 keV
ROC: Receiver operating characteristic curve
AUC: Area under the curve
ROI: Region of interest
DLP: Dose-length product
